# Impact Damage Localization in Composite Structures Using Data-Driven Machine Learning Methods

**DOI:** 10.3390/ma18020449

**Published:** 2025-01-19

**Authors:** Can Tang, Yujie Zhou, Guoqian Song, Wenfeng Hao

**Affiliations:** 1 College of Civil Science and Engineering, Yangzhou University, Yangzhou 225127, China; tangcanvip@126.com (C.T.);; 2 College of Mechanical Engineering, Yangzhou University, Yangzhou 225127, China

**Keywords:** damage localization, composite structures, machine learning, back propagation neural network, arrival time

## Abstract

Due to the uncertainty of material properties of plate-like structures, many traditional methods are unable to locate the impact source on their surface in real time. It is important to study the impact source-localization problem for plate structures. In this paper, a data-driven machine learning method is proposed to detect impact sources in plate-like structures and its effectiveness is tested on three plate-like structures with different material properties. In order to collect data on the localization of the impact source, four piezoelectric transducers and an oscilloscope were utilized to construct an experimental platform for impulse response testing. Meanwhile, the position of the impact source on the surface of the test plate is generated by manually releasing the steel ball. The eigenvalue of arrival time in the time domain signal is extracted to build data sets for machine learning. This paper uses the Back Propagation (BP) neural network to learn the difference in the arrival time of each sensor and predict the location of the impact source. The results demonstrate that the machine learning method proposed in this paper can predict the location of the impact source in the plate-like structure without relying on the material properties, with high test accuracy and robustness. The research work in this paper can provide experimental methods and testing techniques for locating impact damage in composite material structures.

## 1. Introduction

Plate structures, especially composite ones, can be seen everywhere in various fields, such as aerospace and civil engineering [1,2]. The structural health monitoring (Structure Health Monitoring, SHM) method and non-destructive testing (Non-destructive Testing, NDT) technology for these structures are of great significance [3,4,5]. In transportation, service, and maintenance, the plate structure will inevitably bear the impact of various impact objects, such as bird impact, hail impact, tool drops, etc. [6]. The impact can usually be divided into high speed and low velocity [7]. An impact velocity of more than 100 m/s is called high-speed impact. The strain rate is exceptionally high, and it is easy to form apparent damage in the plate-like structure. If the impact velocity is below 10 m/s, it is called low-velocity impact, and the strain rate is low at this time. Phenomena such as falling impacts are all low-speed impacts. This type of damage is often invisible and highly concealed and, to some extent, is more harmful than the more apparent high-speed impact damage. When composite structures are subjected to such impacts, it may cause severe damage to the internal structure without obvious damage marks on the surface, resulting in a relatively low probability of visual detection of damage. If this hidden damage cannot be detected and maintained in time, it may cause disastrous consequences if allowed to accumulate [8]. Currently, the identification methods for low-speed impact load localization mainly include identification methods based on time difference in arrival, impact location identification methods based on optimization algorithms, and impact location identification methods based on signal similarity. Gaul et al. [9] used wavelet transform to extract the difference in arrival time at different frequencies to achieve impact localization on aluminum alloy plates. Sai et al. [10] used the wavelet basis function based on Morlet to extract the wave arrival time difference in the impact response signal. They determined the position of the impact point according to the triangular sensor array. Jiang et al. [11] used the Shannon wavelet to extract the narrowband signal difference in the arrival of the impact response signal. They used the MUSIC algorithm to obtain the position of the impact point. Kundu et al. [12] used six sensors to determine the position of the excitation source on the composite plate without knowing the wave velocity in each direction. Zheng et al. [13] provided an effective strategy for extracting second harmonic Lamb waves from dynamic response signals based on wavelet transforms for the detection of closed microcracks, accurately locating closed microcracks in plate-like metals and composite structures in both numerical simulation and experimental environments. The traditional method based on the time difference in arrival has a certain degree of uncertainty, and it is easy to produce significant errors for the impact localization problem. Seydel [14] roughly calculated the location of the impact load by the time-of-arrival method and then used an optimization algorithm to calculate the minimum difference between the predicted response and the actual response to estimate the impact location. Sai [15] established a nonlinear relationship model between the stress wave time difference in arrival and the spatial position of the sensor. They used the quasi-Newton algorithm to solve the nonlinear equations. The wide application of the optimization algorithm has improved the positioning accuracy very well. Shrestha et al. [16] compared the root mean square error and error outliers of the sample impact point response signal and the unknown impact point response signal to locate the impact of the composite plate structure. Kim et al. [17] achieved the impact localization of composite plate structures by comparing the cross-correlation between the sample point response signal and the unknown point response signal. Zhao et al. [18] performed impact localization based on the k-order natural frequency amplitude deviation between the sample point response signal and the unknown point response signal. Limited by the uncertainty of the material properties of some plate-like structures, its velocity model is often difficult to obtain accurately, leading to deviations in positioning results. Various optimization algorithms can improve accuracy to a certain extent but cannot achieve real-time and fast positioning.

Based on the emerging field of computational intelligence in recent years, new methods exist to process the obtained signals to further improve the accuracy and efficiency of health monitoring of plate structures [19]. Cuomo et al. [20] first obtained a baseline consisting of the structural response induced by the impact test and subsequently evaluated the impact location using the highest cross-correlation coefficient, somewhat overcoming the limitations of the current composite impact localization process. Geng et al. [21] used fiber-grating sensors to obtain structural dynamic response signals and trained neural networks by extracting frequency-domain response features to realize impact damage identification of composite laminates. Using numerical simulation, Dipietrangelo et al. [22] used the K-Fold cross-validation procedure to evaluate the performance of polynomial models of different degrees using different combinations of training/test sets and calculated the average radial error. For shallow neural networks, three learning algorithms were compared: Levenberg–Marquardt, Bayesian Regularization, and Scaled Conjugate Gradient, which confirmed the effectiveness of machine learning for impact detection. Liu et al. [23] used the Empirical Mode Decomposition (EMD) method to remove the trend component in the low-speed impact signal, and extracted time-domain features, frequency-domain features and time-frequency domain features from the preprocessed impact signal, building a Hybrid Support Vector Model to determine the location of low-velocity impacts on composite panel structures. Jiang et al. [24] used fiber Bragg gratings to detect low-velocity impact sources on the surface of carbon fiber-reinforced plastic structures, and used Extreme Learning Machines (ELM) for regional localization. Sai et al. [25] established an Extreme Learning Machine (ELM)-based impact localization model with faster training speed and fewer parameters according to the energy of available frequency band signals. Ai et al. [26] collected an Acoustic Emission (AE) data set by performing impact experiments on a full-scale thermoplastic aircraft elevator in a laboratory environment. A data set consisting of AE parametric features and a data set consisting of AE waveforms were assigned to random forest classifiers and deep-learning networks to study their suitability for impact source localization. Chen et al. [27] utilized a Convolutional Recurrent Encoder-Decoder Neural Network (ED-CRNN) and a Deep Convolutional Recurrent Neural Network (DCRNN) for impact load reconstruction and localization. Jierula [28] installed the sensor in the concrete column, applied the Neighborhood Component Analysis (NCA) feature selection method to select important parameters as the input of the neural network, and proposed a machine learning-based method to locate the impact source in the concrete column. The data-driven method can effectively analyze damage characteristics for classification and identification, overcome the complexity and uncertainty of traditional methods, significantly reduce the workload, and effectively improve the process of structural damage identification. However, complex machine learning algorithms make it difficult to provide the required input parameters in practical engineering testing, resulting in poor engineering practicality. BP neural networks are relatively mature in both network theory and performance. Its outstanding advantages are strong nonlinear mapping ability and flexible network structure. The number of intermediate layers and neurons in each layer of the network can be set arbitrarily according to specific situations, and their performance varies with the difference in structure. However, BP neural networks also have some major shortcomings. The learning speed is slow, and even a simple problem usually requires hundreds or even thousands of learning iterations to converge, therefore, it is easy to fall into local minima. There is no corresponding theoretical guidance for the selection of network layers and the number of neurons. The ability to promote online is limited. There have been many improvement measures for the above issues, with the most researched being on how to accelerate the convergence speed of the network and avoid falling into local minima as much as possible [29,30]. In this paper, the BP neural network is used to detect impact sources in plate-like structures and its effectiveness is tested on three plate-like structures with different material properties.

The follow-up content of this paper is divided into four parts. Section 2 introduces the working principle of the BP neural network used in this paper and the preprocessing of time-domain signals. Section 3 presents the experimental setup and data acquisition system. Section 4 gives the training and prediction results of the model. Section 5 concludes the work.

## 2. Algorithm of BP Neural Network and Signal Preprocessing

### 2.1. The Mechanism of the BP Neural Network

**Figure 1:**
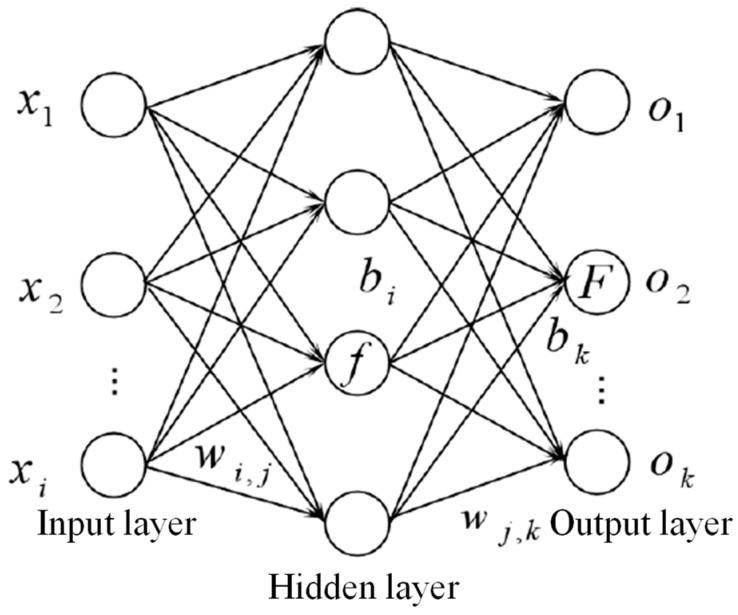
Schematic diagram of BP neural network structure.

The BP neural network is relatively mature in terms of its theoretical basis and performance. Its outstanding advantages are its nonlinear solid mapping ability and flexible network structure. A typical BP neural network consists of three parts: input layer, hidden layer, and output layer, as shown in Figure 1. It has a precise learning mechanism, mainly including two processes: forward propagation of signals and backpropagation of errors. When the input layer of the BP neural network receives the signal, it will pass the signal to the hidden layer, and the hidden layer will process the signal according to the weight, bias, and activation function of the connection, and then pass it to the output layer to output the corresponding predicted value. When the error between the predicted and expected values does not meet the preset target accuracy requirements, the network will feed back the error information layer by layer from the output layer to the input layer. When the error is backpropagated, the weights and offsets of every layer connection will be adapted and updated according to the method of gradient descent. Through continuous training and correction, the error between the predicted and actual values will gradually become smaller, and the predicted result will reach expected outcome. Then, the learning process is over.

The linear function is selected as the activation function of the input layer, and the sigmoid function is used as the activation function of the hidden layer. For the input layer part, the input data are the extracted signal feature value $X={\left[x_{1},x_{2},\ldots ,x_{i},x_{n}\right]}^{T}$, and the output $y_{i}=X_{i}$ due to the use of a linear function.

For the hidden layer part, the input value of the neuron is equal to the sum of the input value connected to it multiplied by the corresponding weight plus the additional bias of the neuron:

(1) $${net}_{j}=\sum_{i=1}^{n}w_{ij}y_{i}+b_{i}$$

n represents the input layer neurons, and j represents the hidden layer neurons.

The output value of the hidden layer is the value calculated by the sigmoid function:

(2) $$y_{j}=f\left({net}_{j}\right)=\frac{1}{1+exp\left(-{net}_{j}\right)}$$

For the input layer part, the input value of the neuron is equal to the sum of the input value connected to it multiplied by the corresponding weight and then the additional bias of the neuron:

(3) $${net}_{k}=\sum_{k=1}^{n}w_{jk}y_{j}+b_{k}$$

Similarly, the output value of the output layer is the value after the sigmoid function operation:

(4) $$y_{k}=f\left({net}_{k}\right)=\frac{1}{1+exp\left(-{net}_{k}\right)}$$

Randomly assign a set of non-zero numbers and small initial values to the weights $w_{ij}$ and $w_{jk}$ between the input layer, the hidden layer, and the output layer. After setting a series of hyperparameters, such as training target, learning rate, the number of iterations, etc., the output $y_{i}$, $y_{j}$, and $y_{k}$ of each layer can be calculated from the output layer. Then, calculate the magnitude of the error between the predicted value and the expected value of each neuron in the output layer:

(5) $$\delta_{k}=\frac{\sum_{k=1}^{n}{\left(y^{\prime }-y_{k}\right)}^{2}}{n}$$

After obtaining the predicted value of the output layer, continue to calculate the error size of each neuron in the hidden layer forward:

(6) $$\delta_{j}=\sum_{j}\delta_{k}w_{kj}$$

Use the resulting error to adjust the weights and biases of each layer:

(7) $$w_{ij}\left(m+1\right)=w_{ji}\left(m\right)+∆w_{ji}\left(m\right)$$

In the following:

(8) $$∆w_{ji}\left(m\right)=m_{c}\cdot w_{ij}\left(m-1\right)+l_{r}\cdot \delta_{j}\cdot f^{\prime }\left({net}_{j}\right)$$

In Equation (7), m is the number of iterations, and in Equation (8), $m_{c}$ is the momentum coefficient; generally 0.9~1. $l_{r}$ is the hyperparameter learning rate.

After a calculation of forward propagation, compare the error $\delta_{k}$ of the output layer with the maximum error ε of the set training target. If $\delta_{k}\leq \varepsilon$, stop training. Otherwise, the error is passed forward, the weights and biases between the layers are adjusted, and the forward propagation is performed again. After the conditions for stopping the training are met, the training ends.

**Figure 2:**
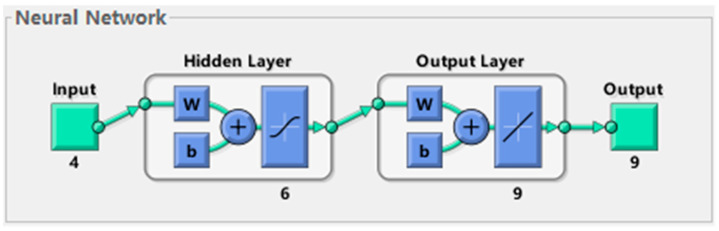
Schematic diagram of the BP neural network model established in this study.

The Sigmoid function requires the input value to be in the interval [−1, 1]. Hence, the samples must be normalized to avoid neuron saturation during training and make the model converge faster. This study uses the mapminmax function for normalization, which can map each sample value to between −1 and 1. The model chosen in this study has a three-tier architecture, including one layer of an input layer, a hidden layer, and an output layer, and its diagrammatic sketch is shown in Figure 2. The programming and training of the BP neural network are carried out on the MATLAB platform. Use the trainlm function as the training function and set the following hyperparameters simultaneously: the maximum training times are 1000, the learning rate is 0.0001, and the minimum error goal is 0.000001. There is no clear theory about determining the number of hidden layer nodes in the neural network. When the nodes’ number is small, it is easy to cause underfitting and affect the prediction accuracy. When the number of hidden layer nodes is too large, the training time of the model may be prolonged, and overfitting is more likely to occur. Here, this study uses the empirical formula: the hidden nodes number = $\sqrt{\left(m+n\right)}+a$ for determining the number of nodes, where m is the amount of input neurons, n is the amount of output neurons, and a is a random number between 1 and 10. Finally, six hidden node numbers are selected according to the formula.

### 2.2. Processing of Captured Signals

**Figure 3:**
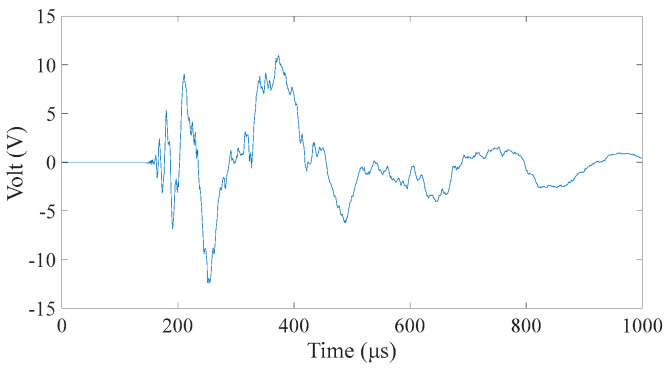
Received signal of a sensor when the aluminum plate is subjected to an impact.

The training of the BP neural network needs to choose a typical characteristic value as the input, so we need to process the time domain signal received by the sensor. Clearly, the position of the sound source strongly correlates with the wave propagation time in the plate. Therefore, we choose the time-of-first-arrival parameter in the time-domain signal. According to the set integration time step and total duration, the length of each sensor signal is 1000 samples, as shown in Figure 3. The time-of-first-arrival of the time-domain signals obtained from the four sensors is collected as the input of the model. It is worth noting that to prevent data leakage during neural network training, this paper performs data segmentation before model evaluation. Then, it performs data preparation only on the training data set and then, applies the data preparation techniques to the training and test sets.

Alt Text: The time-domain signal received by a certain sensor has a signal length of 1000 microseconds. The abscissa of the signal is time in microseconds. The ordinate is the amplitude, and the unit is volts due to the use of an oscilloscope for reception.

## 3. Experimental Procedure

### 3.1. Experimental Setup

**Figure 4:**
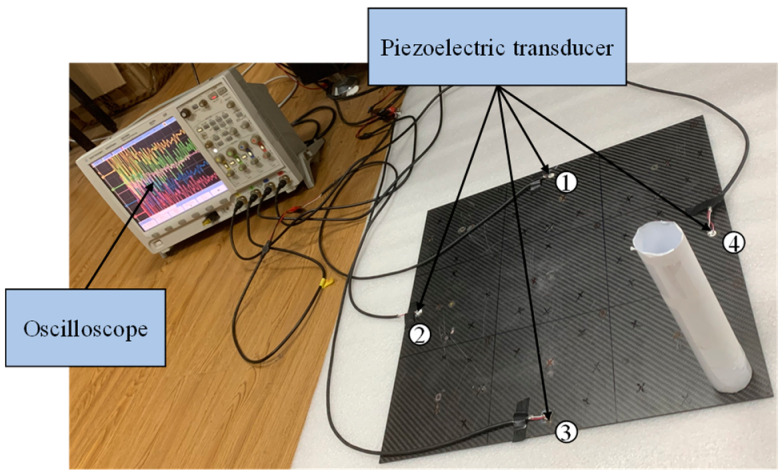
Schematic diagram of the experimental platform.

Generate and record impact signals using the experimental setup shown in Figure 4. The experimental objects in this paper are three different plate structures. The aluminum plate is a representative of isotropic materials, while the unidirectional composite plate and orthotropic plate are typical layering representatives of transversely isotropic materials and composite materials. Among them, the size of the isotropic aluminum plate is $400\times 500\times 1{\;mm}^{3}$, which is divided into nine regions with an area of $133.3\times 166.7{\;mm}^{3}$ on average. The dimensions of the orthotropic laminate and the unidirectional laminate are both $450\times 450\times 3{\;mm}^{3}$, and the thickness of a single layer is 0.125 mm. There are 16 layers in total, equally divided into nine areas. Polymer foam is padded at the bottom of the board to reduce vibration and energy transfer from the environment to the board. A circular piezoelectric transducer (PZT) with a diameter of 10 mm and a thickness of 1 mm is used as the piezoelectric chip, and it is arranged in the middle of the four sides at a distance of 20 mm from the boundary as the receiving source. Before pasting the piezoelectric chip, the surface of the aluminum plate is cleaned with alcohol. Apply the quick-drying adhesive evenly to the designated position and then paste and fix the PZT. A 54820A oscilloscope produced by Agilent was used to record the measurement results. The acquisition signals from all four channels have 16-bit resolution, the sampling rate is set to 1 MHz, the duration of each measurement is 1 ms, and the trigger level is set to 100 mV.

Alt Text: The steel ball is impacted on the surface of the plate structure by paper guides, and the resulting signal is received by piezoelectric sensors on the four edges of the plate and displayed by an oscilloscope.

Impact signals are generated by impacting pellets of different diameters. Steel balls made of hardened steel excite elastic waves in the plate, which are released from different heights. The diameters of the respective balls were 8 mm, 10 mm, and 15 mm. The repeatability of the impact position and height is ensured by a paper guide, which determines the orientation of the steel ball during free fall. The thin plate entirely absorbs the impact energy of the steel ball, and elastic deformation mainly occurs during the impact process. The acquired sensor signal shows only a single waveform, which is excited by the ball’s impact. The lack of other waveforms in the captured time-domain signal indicated that the rebound of the steel ball was not recorded. The model’s training needs to input a certain number of samples with specific variability. The resulting waveform after each impact is differently affected by damping, dispersion, and edge reflections, allowing us to obtain a highly variable database.

### 3.2. Experimental Process

**Figure 5:**
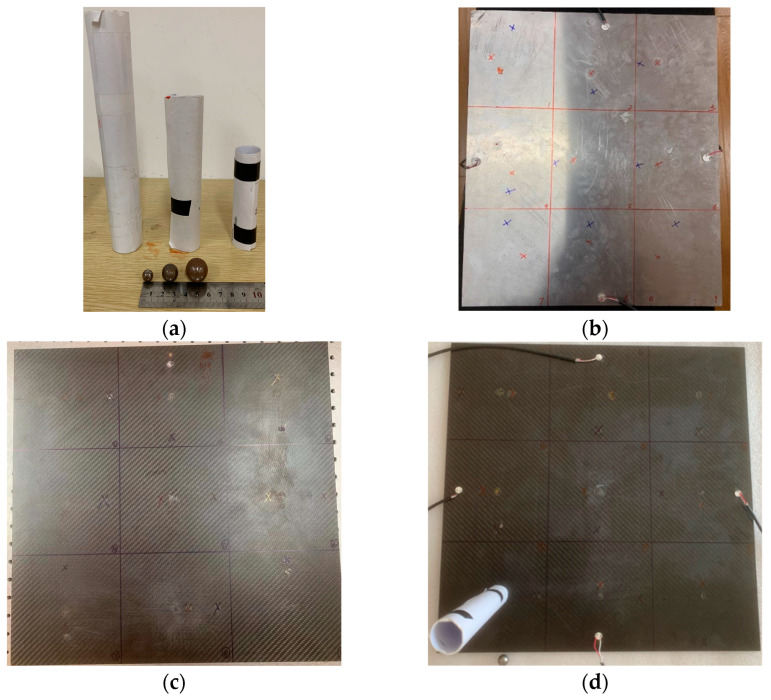
Schematic diagram of different test sets (red marks are 30 mm and 20 mm from the center of the area, and blue is 60 mm): (**a**) balls with different diameters and guide rails with different lengths; (**b**) schematic diagram of the impact position of the aluminum plate; (**c**) schematic diagram of the impact position of the orthotropic laminate; (**d**) schematic diagram of impact position of unidirectional laminate.

This paper tests the influence of three factors on the accuracy of the BP neural network in predicting the impact area of the falling ball. The experimental procedure is as follows:

(1) At the center points of nine areas, use a small steel ball with a diameter of 10 mm to drop from a height of 200 mm; repeat ten times for each area center, and a total of 90 sets of data are utilized as the training set of the model.
(2) Explore the impact of different drop heights on prediction accuracy. Also, use 10 mm small steel balls to fall at the center points of each area, change the length of the guide rail, and make the small balls fall from the heights of 150 mm and 100 mm, respectively, as shown in Figure 5a. Each regional center is repeated ten times, and a total of 90 sets of data are used as the test set of the neural network.
(3) Explore the influence of different steel ball diameters on the prediction accuracy. Also, use a 200 mm long guide rail at the center point of each area to ensure that the steel balls fall at the same height, change the diameter of the small balls used, and use small steel balls with diameters of 8 mm and 15 mm to drop from the same height, as shown in Figure 5a. Each regional center is repeated ten times, and a total of 90 sets of data are utilized as the test set of the model.
(4) Explore the impact of different falling positions on prediction accuracy. Steel balls of the same diameter as the test set were dropped from the same height. For the aluminum plate, offset the drop point by 30 mm and 60 mm from the center of the area. For two composite material panels, the landing point positions are randomly selected at 20 mm and 60 mm from the center of the area. As shown in Figure 5. Each drop point is repeated ten times, and 90 sets of data are used as the test set.

Alt Text: The three plate-like structures used in this paper were all divided into nine identical areas, and each area was marked with a marker pen. The red mark is 20 mm from the center of the area, and the blue mark is 60 mm from the center of the area.

## 4. Prediction Results of Impact Source Localization

### 4.1. Prediction Results of Impact Source Localization in Aluminum Plate

**Figure 6:**
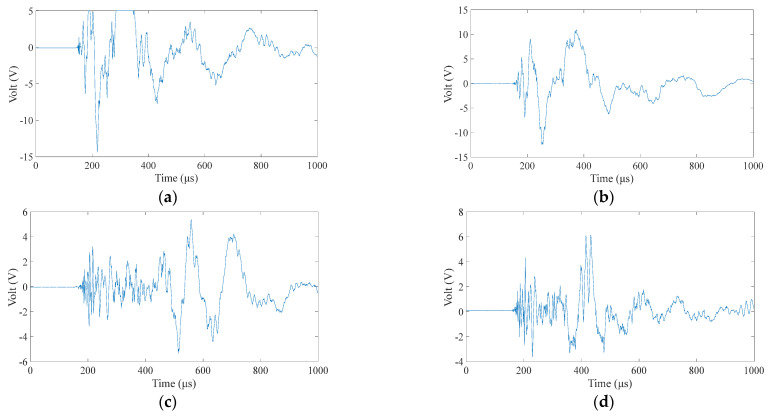
The signals received by the four sensors during a fall in area 1 of the aluminum plate. (**a–d**), respectively, correspond to No. 1–4 piezoelectric sheet (PZT) in Figure 4.

**Figure 7:**
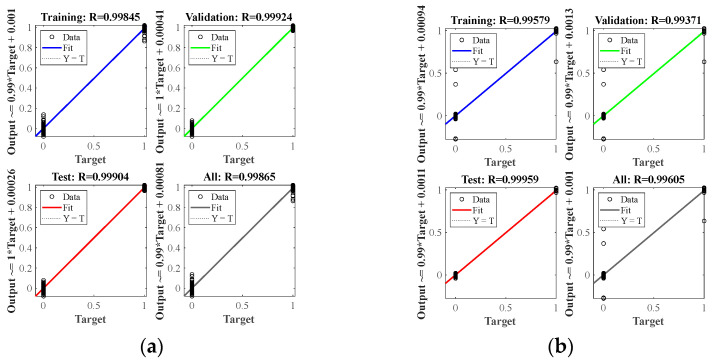
Training results for different test sets in aluminum plates: (**a**) test sets for different steel ball diameters and dropping heights; (**b**) test sets for different impact positions.

At nine center points of the area, a small steel ball with a diameter of 10 mm was dropped from a height of 200 mm, each area center was repeated ten times, and a total of 90 samples were used as the training set. It can be seen from Figure 6 that in the isotropic plate, due to the different positions of the sensors, there are apparent differences in the arrival time of the wave generated by the impact source to the four sensors. A neural network model can learn this difference and map the arrival times of the four sensors to the source of the impact. After preprocessing the time of arrival of each time-domain signal as described in Section 2.2, input it into the BP neural network described in Section 2.1 for training. After the training is completed, collect the test set data described in Section 3.2, and input the neural network as the test set according to the same steps. Figure 7 shows the training results of two different data sets when predicting. The correlation coefficient R in the figure indicates the degree of fitting of the data. It can be seen from the figure that the correlation coefficients of all groups in the aluminum plate are above 0.99, the fitting accuracy is high, and the model performs well. At the same time, the fitting degree of the training set and the test set is very high, and there is no over-fitting phenomenon, which also benefits from the relatively simple structure of the BP neural network.

Alt Text: There is a significant difference in the arrival time of the waveforms of the signals received by the piezoelectric sensor located at the midpoint of the four sides after an impact in the aluminum plate. The arrival time of the waveform is extracted as the input of the neural network.

Alt Text: The training results of each test are set in the aluminum plate experiment. The horizontal and vertical coordinates show the error between the expected value and the actual value, and the fitting coefficient shows the fitting accuracy of each group.

**Figure 8:**
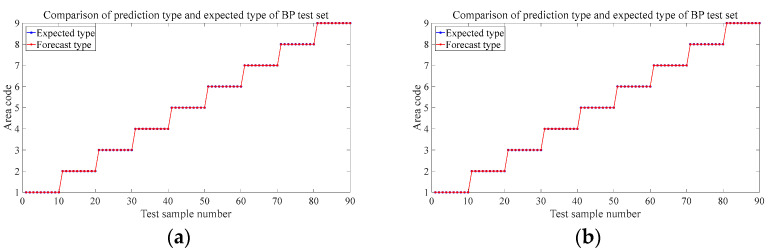

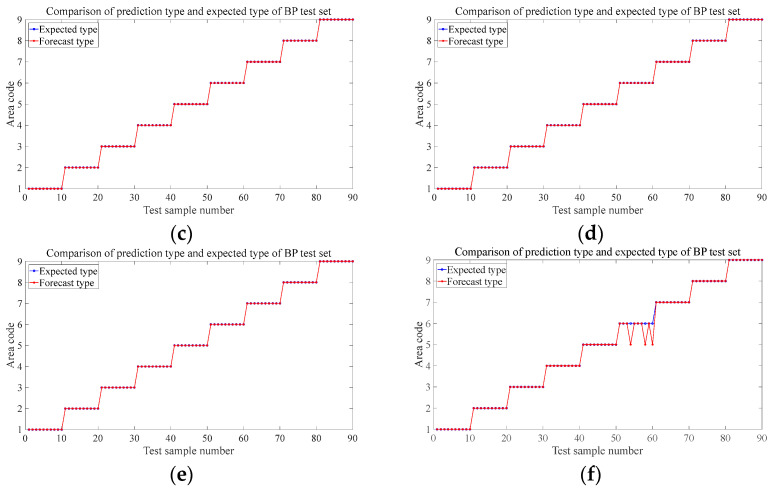
Prediction results for different test sets in aluminum panels. (**a**) The test set at 150 mm in 3.2 (2). (**b**) The test set at 100 mm in 3.2 (2). (**c**) The test set at 8 mm-diameter steel balls in 3.2 (3). (**d**) The test set of 15 mm-diameter steel balls in 3.2 (3). (**e**) 3.2 (4). The test set at 30 mm from the center of the area. (**f**) The test set at 60 mm away from the center of the area in 3.2 (4).

Figure 8 shows the prediction results of the six test sets in the aluminum plate. The blue circle in the figure represents the expected value, that is, the area where the steel ball falls in the experiment, and the red represents the discriminant result of the falling area by the trained neural network based on the eigenvalues input from the test set. It can be seen from the results that the prediction accuracy rate of the first four groups is 100%, and different drop heights and different steel ball diameters do not affect the prediction accuracy. The trained BP neural network can very accurately judge the area where the steel ball falls at this time and has a strong robustness to these two influencing factors. Figure 8e shows that the neural network model trained by the data sets of each regional center can accurately predict the location of the impact source within 30 mm nearby with 100% accuracy, which has specific prospects for practical engineering applications. When we change the training set’s position to 60 mm, the position of the impact is close to the area’s boundary. Figure 8f shows that the prediction accuracy is 96.7%, and only three misjudgments occur, which is still a more precise result. These three misjudgments were all misjudgments from area 6 to area 5. It can be seen from the blue mark in Figure 5b, the impact point of the steel ball at this time is close to the boundary of these two areas, so a wrong prediction is produced.

Alt Text: The prediction results of each test set in the aluminum plate experiment. The blue markers represent the expected value, and the red markers represent the output of the neural network. The abscissa represents the sample number of the test set, and the ordinate represents the area code of the output. In the test results of different steel ball diameters and different drop heights, the red mark and the blue mark completely coincide, which means that the prediction accuracy is 100%. There were three wrong predictions in the test set where the impact location was close to the region border.

**Figure 9:**
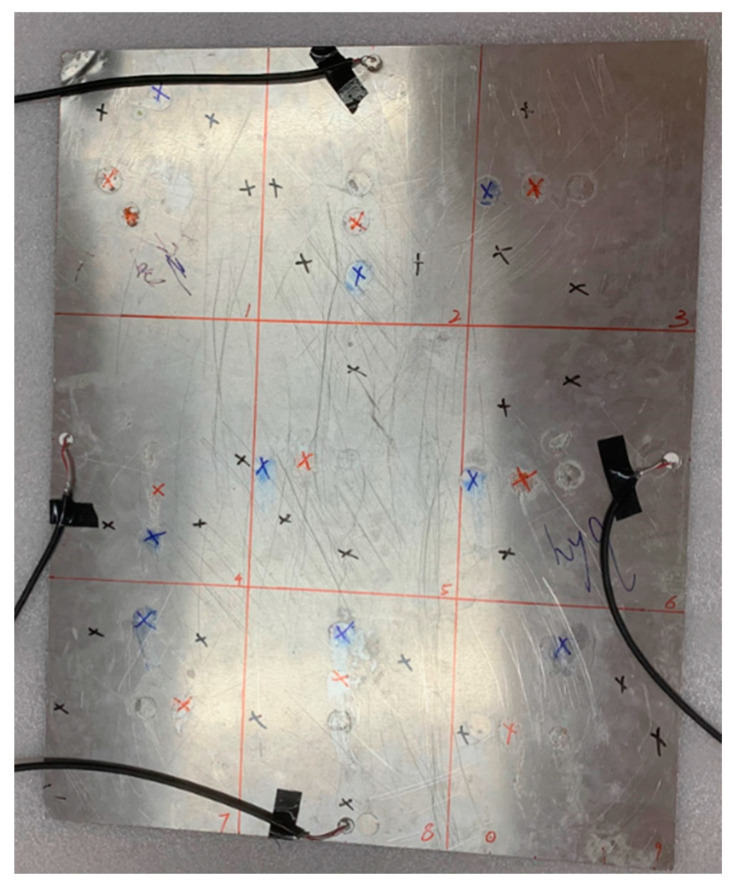
Schematic diagram of the increased impact location on the surface of the aluminum plate. The distance between the black and blue marks is 60 mm from the center of the area.

**Figure 10:**
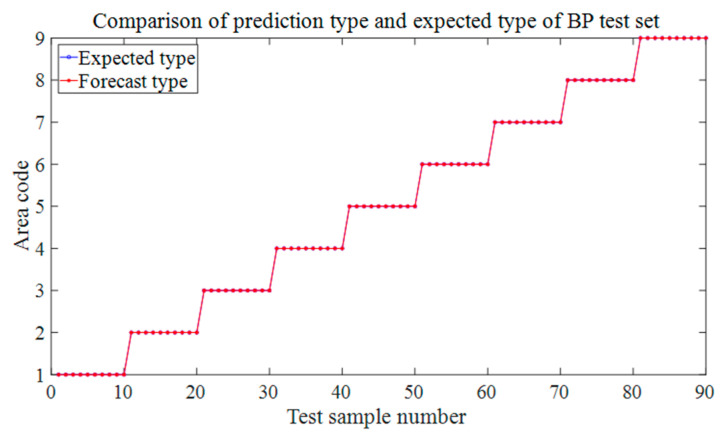
Prediction results after expanding the training set.

Because of the situation in Figure 8f, this paper tries to improve the prediction accuracy by expanding the training set. Specifically, as shown in Figure 9, continue to take three points at the same distance of 60 mm from the center of the area and mark them as black. Drop a steel ball with the same diameter from the same height at the black mark and repeat 10 times for each. In this way, the training set of each region is increased by 30, and the entire training set is expanded to 360. After expansion, the neural network is retrained, and the 90 sets of data marked in blue are also used as the test set. The prediction results at this time are shown in Figure 10. When the types of training sets were expanded, the neural network newly learned the characteristics of the arrival time when the impact source was 60 mm from the center of the area, thus improving the accuracy of this part of the test set, reaching 100% like other test sets.

Alt Text: On the basis of Figure 6a, continue to use a black marker to mark at the same distance from the center of the area 60 mm. The new sampling points will be used as the expansion of the training set to input the neural network.

Alt Text: The prediction results after adding the samples marked in black in Figure 10 to the training set. The prediction results in Figure 8f are improved, so that the three wrongly predicted samples are correct again.

### 4.2. Impact Source Localization Results in Composite Panels

**Figure 11:**
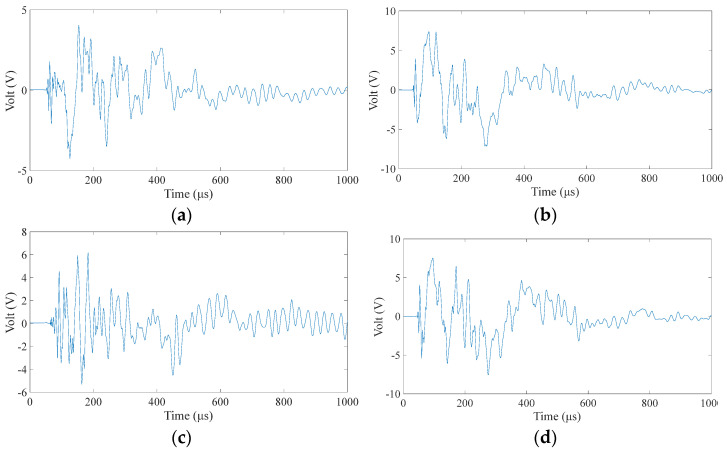
Signals received by four sensors during a fall in area 1 in an orthotropic laminate. (**a**–**d**), respectively, correspond to No. 1–4 PZT in Figure 4.

**Figure 12:**
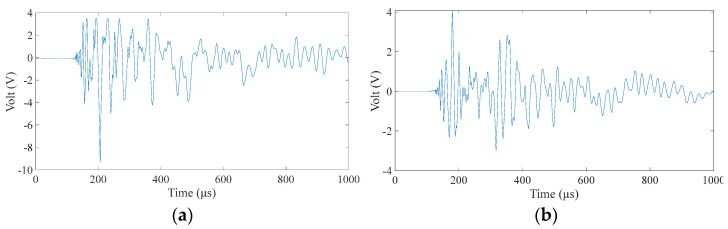

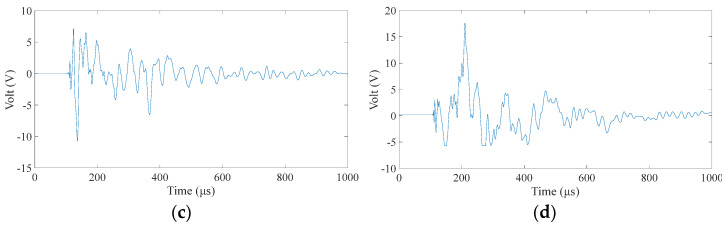
Signals received by four sensors during a fall in zone 1 in a unidirectional laminate. (**a**–**d**), respectively, correspond to No. 1–4 PZT in Figure 4.

**Figure 13:**
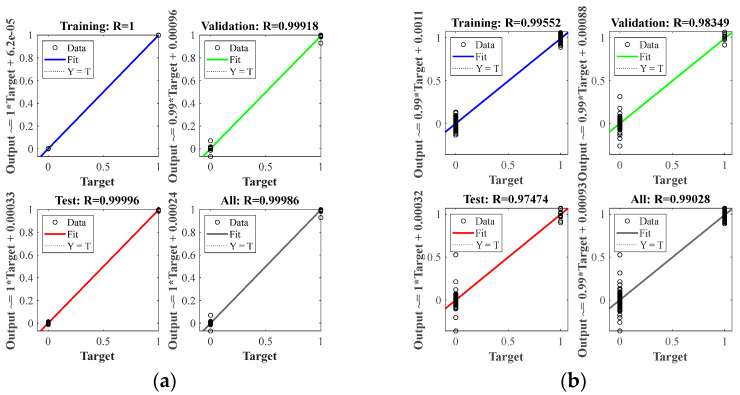
Training results for different test sets in orthotropic laminates: (**a**) test sets for different steel ball diameters and dropping heights; (**b**) test sets for different impact positions.

**Figure 14:**
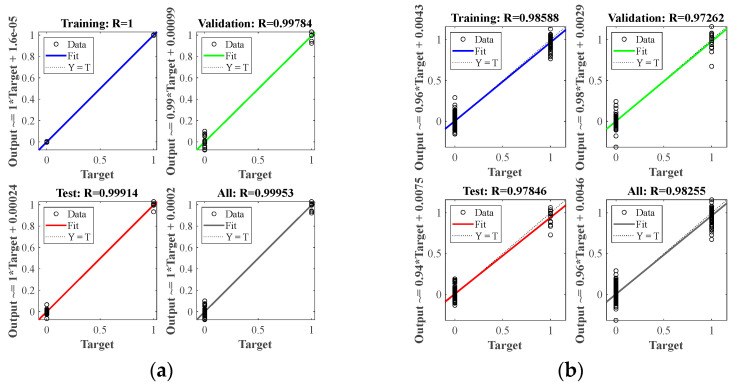
Training results for different test sets in unidirectional laminates: (**a**) test sets for different steel ball diameters and dropping heights; (**b**) test sets for different impact positions.

The wavefield information in the composite slab was collected using the same experimental platform as in Figure 4. This paper uses two types of carbon fiber-reinforced composite laminates: orthotropic and unidirectional laminates. The geometric parameters are described in Section 3.1. Due to the difference in lay-up direction, the propagation mode of the wave in the two plates is also different, and both are different from the isotropic aluminum plate. This section aims to test whether the neural network can still map arrival times to impact sources under different propagation modes. Figure 11 and Figure 12 show the signals received by the sensors for a ball impact at the center of zone 1 in two panels. Although the steel ball impact is also performed in area 1, the signals captured by the piezoelectric sheets are also different due to the different laying directions of the composite materials. There are apparent differences in the arrival times of the three plates. Traditional methods based on the time difference in arrival often require accurate material properties, so it is difficult to achieve fast and accurate positioning for composite material panels with uncertain material properties. Perform data processing and network testing the same way as in the aluminum plate experiment. Set the same training set and test set according to Section 3.2. The training results of different test sets are shown in Figure 13 and Figure 14. The fitting degree of each data set in the two composite material plates is good. The test is set at 60 mm from the center of each area. Both of them have relatively low correlation coefficients, 0.97474 and 0.97262, respectively, which may affect the model prediction accuracy, but is still an acceptable result. No overfitting was observed.

Alt Text: The signals received by the four sensors during an impact at the center of zone 1 in an orthotropic laminate. Compared with the signal in the aluminum plate in the previous section, there are obvious differences in the arrival time of the waveform, and the relationship between different arrival times is no longer linear, the wave speed is faster, and the arrival time values are smaller.

Alt Text: The signal received by the sensor when the central position of area 1 in the unidirectional laminate receives an impact. Although the impact is also performed in area 1, the signals captured by the piezoelectric sensor are also different due to the different laying directions of the composite material. The wave velocity in unidirectional laminates is between aluminum and orthotropic laminates, and the wave velocity along the ply direction is relatively fast.

Alt Text: Training results for each set of test sets in orthotropic laminates. A similar fitting accuracy to the data set in the aluminum plate was achieved.

Alt Text: Training results for each set of test sets in unidirectional laminates. The fitting accuracy of each test set is above 0.97.

Alt Text: The prediction results of each test set in the orthotropic laminate experiment. In the test results of different steel ball diameters and different drop heights, the red mark and the blue mark completely coincide, which means that the prediction accuracy is 100%. There were four wrong predictions in the test set where the impact location was close to the region border.

Alt Text: The prediction results of each test set in the unidirectional laminate experiment. In the test results of different steel ball diameters and different drop heights, the accuracy rate is also 100%. There were four wrong predictions in the test set where the impact location was close to the region border, they both appeared in area 4 and were misjudged to area 7.

**Figure 15:**
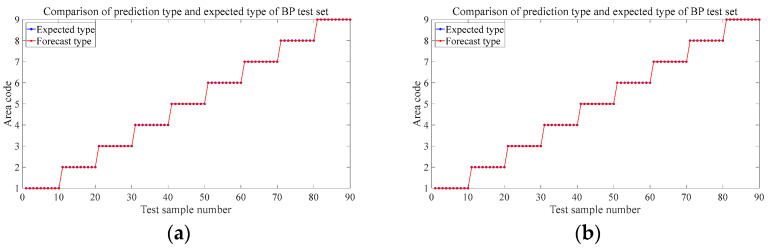

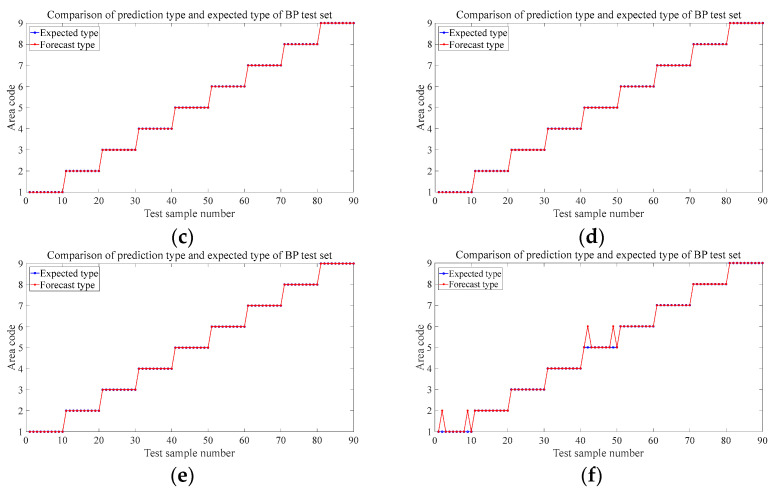
Prediction results for different test sets in orthotropic laminates. (**a**) The test set at 150 mm in 3.2 (2). (**b**) The test set at 100 mm in 3.2 (2). (**c**) The test set at 8 mm-diameter steel balls in 3.2 (3). (**d**) The test set at 15 mm-diameter steel balls in 3.2 (3). (**e**) 3.2 (4) The test is set at 30 mm from the center of the area. (**f**) The test set at 60 mm away from the center of the area in 3.2 (4).

**Figure 16:**
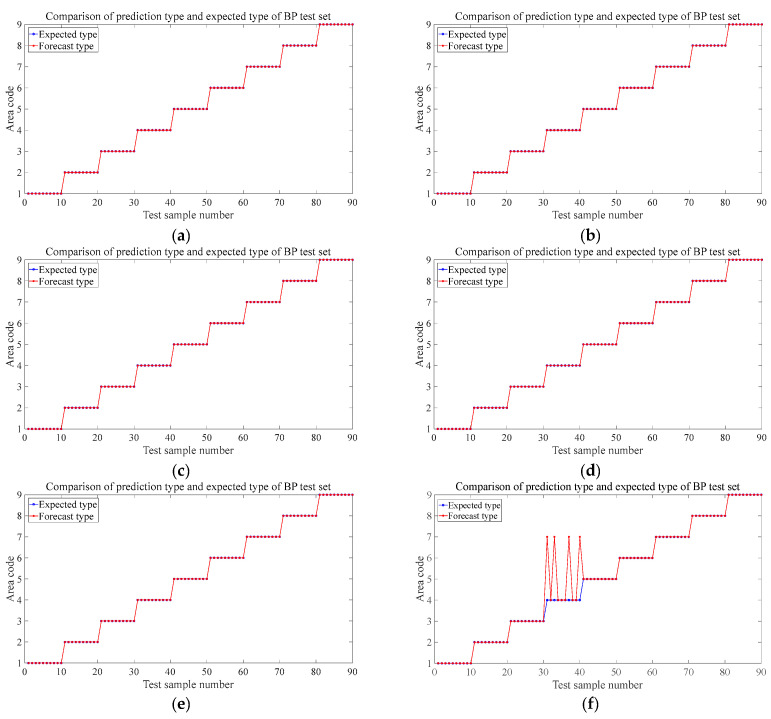
Prediction results for different test sets in unidirectional laminates. (**a**) The test set at 150 mm in 3.2 (2). (**b**) The test set at 100 mm in 3.2 (2). (**c**) The test set at 8 mm-diameter steel balls in 3.2 (3). (**d**) The test set at 15 mm-diameter steel balls in 3.2 (3). (**e**) 3.2 (4) The test set at 20 mm from the center of the area. (**f**) The test set at 60 mm away from the center of the area in 3.2 (4).

**Figure 17:**
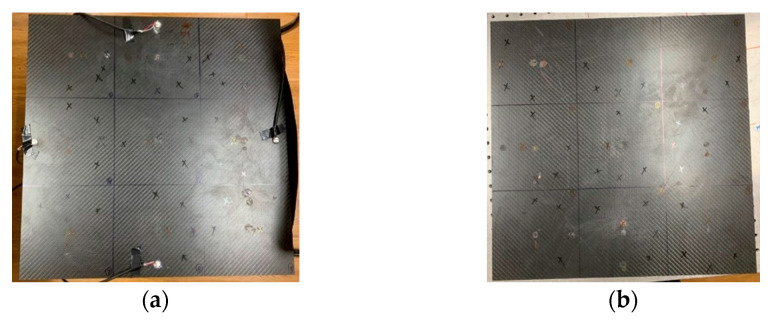
Schematic diagram of the location of the added training set sampling points: (**a**) orthotropic laminates; (**b**) unidirectional laminates.

**Figure 18:**
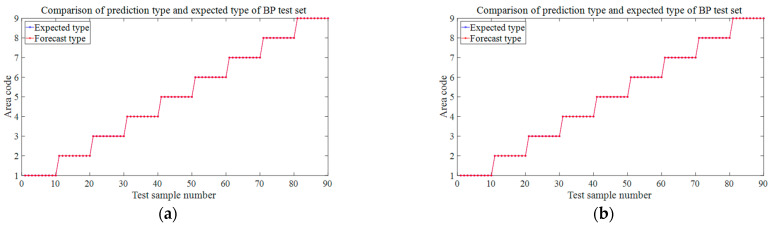
Prediction results of each plate after expanding the training set: (**a**) schematic diagram of increased sampling points of orthotropic laminates; (**b**) schematic diagram of sampling points added to unidirectional laminates.

Figure 15 and Figure 16 are the prediction results of the trained model for the location of the impact source in two composite laminates. The prediction results of the test set at different drop heights, different steel ball diameters, and 20 mm near the center of the area are the same as the aluminum plate and also have strong robustness, and the prediction accuracy reaches 100%. The prediction accuracy of the steel ball drop position test set at 60 mm from the center of each area is slightly lower than that of the aluminum plate, both of which are 95.6%, and four wrong predictions are different from the expected results. In the orthogonal plate, two samples of the test set in region 1 were mispredicted to region 2; mispredictions also occurred in region 5, and 2 samples were judged to belong to region 6. The four mispredictions in the one-way board all occurred in area 4 and were misjudged in area 7. Combined with the blue marks in Figure 5c,d, it can be seen that because they are very close to the boundaries of each region, the samples with wrong predictions are all mistaken by the network as the impact source of the adjacent region. Like the aluminum plate in Section 4.2, this study also tries to expand the training set to improve prediction accuracy. Figure 17 is the newly added impact position of the falling ball. Three black marks are added in each area of the two laminated plates. All of them are 60 mm away from the center of each area. A small steel ball with a diameter of 10 mm is dropped from 200 mm at each mark, repeated 10 times. This increases the number of samples to train the neural network to 360. Figure 18 shows the predicted results at this time. Similarly to the aluminum plate experiment results, after increasing the diversity of the training set, the model can predict with 100% accuracy the area where the impact source of the small steel ball belongs.

Alt Text: On the basis of Figure 5c,d, continue to use a black marker to mark at the same distance from the center of the area 60 mm.

Alt Text: The prediction results after adding the samples marked in black in Figure 18 to the training set. The prediction results in Figure 16f are improved, the respective for wrong samples were re-predicted as correct.

## 5. Conclusions

This paper uses a machine learning approach to locate the source of a falling ball impact on the surface of three panel-like structures with different material properties, including an isotropic aluminum panel and two composite laminates with different ply orientations. In this study, the impact sources are from signals at different positions of the piezoelectric sensor at different times. The most typical three-layer BP neural network is utilized to learn this feature. When the material properties are unknown, four sensors are used to test the low-velocity impact source of the steel ball through regional targeting. A training data set is generated in the center of each area, and the area in which the impact sources in other locations belong is predicted. The robustness of the model is tested at the same time. Experiments were carried out on three plate structures with different material properties to verify the proposed scheme. The results reveal that the trained model can precisely predict the area where the impact source is located, whether in isotropic aluminum panels or composite panels with different ply directions. At the same time, the prediction result has strong robustness. Changing the diameter and drop height of the falling steel ball within a specific range does not affect the prediction accuracy (100%). When the falling position of the steel ball is 20 mm or 30 mm away from the center of the area where the training set data are located, all the training samples in the three boards can accurately determine the area they belong to, with an accuracy rate of 100%. When the drop position continues to be away from each center point to 60 mm, the prediction result in the aluminum plate is better, with an accuracy rate of 96.7%. The accuracy within the two composite panels was slightly lower at 95.6%. In addition, a method of augmenting the training set is adopted to improve the case of misassignment of shocks at the boundaries of each region. After adding samples at the same distance of 60 mm from the center as the training set, the model’s performance improved, and the prediction accuracy reached 100%. The above results demonstrate the potential of the method proposed in this study in effectively characterizing the impact source locations on the surface of plate-like structures. This paper did not consider the impact of the environment and the anti-interference ability of the proposed model. Our future research will focus on discussing the impact of environmental factors such as noise and temperature on the results.

## Data Availability

The original contributions presented in this study are included in the article. Further inquiries can be directed to the corresponding author.
